# Chemometric Screening of Fourteen Essential Oils for Their Composition and Biological Properties

**DOI:** 10.3390/molecules25215126

**Published:** 2020-11-04

**Authors:** Filomena Monica Vella, Roberto Calandrelli, Domenico Cautela, Immacolata Fiume, Gabriella Pocsfalvi, Bruna Laratta

**Affiliations:** 1 National Research Council (CNR), Institute of Research on Terrestrial Ecosystems (IRET), via P. Castellino, 111-80131 Naples, Italy; filomenamonica.vella@cnr.it (F.M.V.); roberto.calandrelli@cnr.it (R.C.); 2 Experimental Station for the Industry of the Essential Oils and Citrus Products (SSEA)-Special Agency of the Chamber of Commerce of Reggio Calabria, via T. Campanella, 12-89125 Reggio Calabria, Italy; dcautela@ssea.it; 3 National Research Council (CNR), Institute of Biosciences and BioResources (IBBR), via P. Castellino, 111-80131 Naples, Italy; immacolata.fiume@ibbr.cnr.it

**Keywords:** essential oils, PCA analysis, GC–MS chemotyping, antioxidant activity, antiyeast activity

## Abstract

Essential oils (EOs) obtained from aromatic plants are widely used worldwide, especially in cosmetic and food products due to their aroma and biological properties and health benefits. Some EOs have significant antimicrobial and antioxidant activities, and thus could effectively increase the shelf lives of foodstuff and beverages. In this study, fourteen essential oils (clove, eucalyptus, fennel, lavender, oregano, palmarosa, pepper, star anise, tea tree, turmeric, Chinese yin yang, Japanese yin yang, and ylang ylang) from different medicinal plant families were screened by gas-chromatography–mass spectrometry (GC–MS) for their different chemical profiles and bioassays were performed to assess their antifungal and antioxidant activities. The results obtained were assessed by principal component analysis (PCA). PCA distinguished six groups characterized by different terpene chemotypes. Amongst the EOs studied, the clove EO showed the strongest antioxidant activity characterized by an EC_50_ of 0.36 µL/mL. The oregano EO had the greatest antiyeast activity characterized by a minimal inhibitory concentration of 10 µL/mL. In conclusion, clove and oregano EOs are strong antifungal and antioxidant agents, respectively, with great potential in the food industry to avoid spoilage and to increase shelf life.

## 1. Introduction

Aromatic plants (APs) have been used since antiquity in folk medicine and as food preservatives due to their many biologically active components including phenolic compounds (e.g., phenolic acids, flavonoids, coumarins, lignans, stilbenes, and tannins), nitrogen-containing compounds (alkaloids, amines, and betalains) and terpenoids (including carotenoids). Essential oils (EOs) are the volatile fractions extracted from aromatic plants. EOs are highly complex natural mixtures of low-molecular mass volatile compounds such as terpenes and aldehydes that are responsible for the typical aroma of any APs [1,2,3,4,5,6]. EOs are produced by the different parts of plants, including buds, flowers, leaves, stems, twigs, seeds, fruits, roots, wood and bark, and stored in secretory cells, cavities, canals, epidermic cells and glandular trichomes [1,2,4,5,6]. Nearby 3000 EOs originated from 2000 plant species and are distributed around 60 botanical genera which are produced worldwide. In nature, EOs play important biological and ecological roles since they are directly involved in plant communication and thus are considered as the language of chemical signaling [7]. In plant defense, they act as antibacterial, antiviral, antifungal and insecticide agents. Additionally, some EOs may attract certain insects to promote the dispersion of pollens and seeds, or to repel undesirable others [3,7]. The term essential oil was used for the first time in the 16th century by Paracelsus von Hohenheim, who referred to the effective component of a drug as “Quinta essential” [8]. EOs have widely been used for centuries for their well-known virucidal, antibacterial, antifungal, anticancer, antioxidant, and antidiabetic activities in medicinal and pharmaceutical formulations, in perfumery, fragrance, and cosmetic industries, in aromatherapy and as food additives [1,2,3,4,6,9].

Traditionally the use of EOs is always narrow and limited to aromatherapy (baths, massages and wraps) and cosmetic applications (body creams, lotions and masks). Recently they have received considerable interest as new functional food ingredients. In fact, EOs are increasingly applied in foods and drinks which is boosted by a rising interest of consumers in natural supplements and the growing concern about potentially harmful synthetic additives [3,6,7]. Moreover, today many of the EOs are classified as Generally Recognized as Safe (GRAS) substances which makes them invaluable for use in food preparation, drugs, and cosmetics [2,10,11,12]. Many APs find their ways as resources of high-end value drugs and lead compounds too. Today there are a few hundred of EOs that are commercially important [1,3,5,7]; many are used in the medicinal and cosmetic fields, and in the food packaging industry.

Fresh and stored food, such as raw olive, dairy products (cheeses and yogurts), fruits and products (jams, canned fruits, dried fruits, glacéd fruits, and fruit salads), bakery products, alcoholic (wine and beer) and non-alcoholic (juice and soft drinks) beverages can undergo microbial contamination [13,14]. Food spoilage by microorganisms leads to significant quality deterioration and results in in huge economic losses [2]. In order to ensure the quality and safety of food products and thus to protect consumer health, effective antimicrobial and antioxidant measures are necessary. Heat treatment, pasteurization, and antiseptic packaging are the most used methods to avoid microbiological deterioration of fruit and vegetable juices and purees and to exclude spoilage as long as the packaging remains intact [15]. Spoilage is often a consequence of yeast growth in food and beverages leading to turbidity, sedimentation, gassiness and off-flavor [16]. *Saccharomyces cerevisiae* is widely found in the industrial fermentation of foods and beverages containing mono- and disaccharides. Common cases of food spoilage by yeast are reported in fresh sea food, packed meat, fresh and frozen vegetables, wines and dairy products. Foods and drinks that cannot be pasteurized are usually treated with preservatives to prevent the growth of mold, yeast and fungi. Primarily used chemical preservatives are weak acids, such as sorbic and benzoic acid, and their salts or sulfites. Recent studies, however, raise concerns about the use of some chemical preservatives such as benzoic acid that can degrade to benzene, a carcinogen substance when used in foods [15,17]. Further, to avoid fungal growth on crops, spraying fungicides over the fields is a frequently used practice. However, most of the fungicides as well as their degradation products can contaminate the foodstuff and thus their use is an issue for food safety.

In general, EOs display their antiyeast, antimold and antifungal activities by means of irreversible damages affecting the membrane permeability and osmotic balance of the cell. Furthermore, they were reported to be able to modify ion transport processes and interact with vital cellular constituents including membrane proteins, resulting in water imbalance, depletion of intracellular ATP concentration and finally causing cell death [2,18,19,20]. Furthermore, EOs possess antioxidant activity capable of counteracting the action of free radical-mediated lipid peroxidation with the resulting decrease in shelf stability of foodstuffs [21,22,23,24]. Most prominently, terpenes, terpenoids and phenylpropanoids, having lipophilic nature and showing remarkable antimicrobial activity, are the main active ingredient of EOs.

Here, we studied fourteen widely used EOs (clove, eucalyptus, fennel, lavender, oregano, palmarosa, pepper, star anise, tea tree, turmeric, Chinese yin yang, Japanese yin yang, and ylang ylang) extracted from five different medicinal plant families: *Apiaceae, Lamiaceae, Myrtaceae, Poaceae* and *Rutaceae*. These medicinal plant families are of considerable interest because they are rich in phenylpropanoids [1,2]. Volatile phenylpropanoids have multifaceted effects, which include antimicrobial and antioxidant properties. Thus, antiyeast and antioxidant activities alongside with their chemical compositions of the above EOs were investigated in order to evaluate their potential applicability as natural preservative agents in food and cosmetic industries, as well as in the human health field.

## 2. Results and Discussion

### 2.1. EOs Chemical Composition and PCA Analysis

The chemical composition of the 14 EOs was determined by gas-chromatography–mass spectrometry (GC–MS) analysis. Table 1 shows the list of compounds in order of their elution time. A total of 177 compounds were detected, with a number of identified molecules ranging between 7 and 62 for the individual EO. The most abundant components belong to the monoterpenoids and sesquiterpenes. In the clove EO, we detected fewer components, while the pepper EO was found to be the richest one, with 62 compounds identified.

The tea tree EO was rich in terpinen-4-ol (47.5%) and γ-terpinene (17.2%), in accordance with data reported by Noumi et al. [25] and Zhang et al. [26]. Star anise and fennel EOs had anethole as their main component, with values of 75.0% and 50.8%, respectively [4,27,28]. d-limonene was particularly abundant in the terpene profile of the bitter orange EO with a percentage of 93.9% [29,30]. Moreover, the pepper EO extracted in our laboratory showed a percentage of d-limonene of 21.3% similar to o-cymene, which represented 23.9% [31,32]. EOs eucalyptus and Chinese yin yang had eucalyptol as the most abundant compound—88.3% and 34.5%, respectively [23,25]. For the mixed Chinese yin yang, the results showed that is mainly consisted of EO from *Eucalyptus globulus*. The terpene profiles of the turmeric, clove, oregano and palmarosa EOs were found to be similar to those present in the literature data. In particular, turmeric was mainly composed by 29.3% of *ar*-tumerone and 28.8% of α-turmerone [23], and clove was made up of eugenol with a percentage of 82.7% [6,22,33,34]. Furthermore, the oregano EO had 69.6% carvacrol [6,33] and palmarosa EO had 82.6% geraniol [35]. The Japanese yin yang EO contained menthol (35.6%) and l-menthone (33.8%), characteristic molecules of the *Mentha arvensis* plant from which the EO was already characterized [36,37]. Ylang ylang EO showed β-copene (19.5%) and caryophyllene (15.5%) and this chemotype was different from that reported in other studies in the literature [23]. The differences in terpene composition could be due to the intrinsic characteristics of plant such as age, stage of development, degree of maturity, and genetic variability, from which the EO used in the present study was extracted. Moreover, it is well-documented that environmental conditions (climate, soil, altitude, latitude, etc.) and the method used for the extraction affect the chemical composition of EOs [1,7]. Finally, the lavender EO was mainly composed of the monoterpene linalool (23.3%) and of the *p*-anisaldehyde at 30.3% which was for the first time reported in lavender EO, unlike what has been described in the literature so far [38,39,40].

We found that the GC–MS profile was characteristic to each EO. The chemical profile obtained by GC–MS analysis (Table 1) contains quantitative data of the different plant metabolites present in the EOs that enables their molecular-based classification, which can be explored by means of multivariate statistical techniques. Here principal component analysis (PCA), one of the most widely used techniques, was used to elaborate possible relationships between EOs and their terpenic composition [41,42]. PCA reduces the complexity of original data meanwhile retaining most of the information (variables) to emphasize variation and bring out strong patterns in a dataset. Recently PCA has been shown to be very useful when combined with targeted and untargeted analytical fingerprinting techniques and applied to herbal extracts and EOs [43].

**Figure 1:**
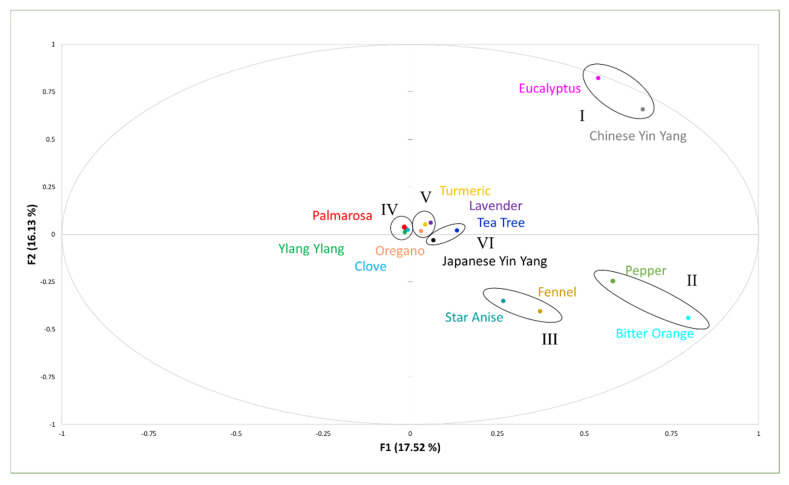
Principal component analysis (PCA) involving compositions of 14 EOs determined by gas-chromatography–mass spectrometry (GC–MS).

In this work, chemotyping of EOs was performed by studying the chemical variability between the samples and transforming the original correlated variables into new, reduced, artificial, and orthogonal ones, called principal components (F1 and F2 in Figure 1). The score-plot shows the relationship between different EOs (the observations) and the loading plot shows how strongly each characteristic influences a principal component (other variables). Graphically, EOs close to each other in the score-plots have similar chemotype. In this regard, the first two principal components (F1 and F2) had the highest share of the total variance (33.6%), due to the relatively high number of variables analyzed (177 compounds). In fact, twelve principal components would explain 100% of the total variation. It is, however, worth noting that the number of principal components depends on the number of variables, as the percent of total variance increases with lower number of variables. Generally, by increasing the number of variables, the proportion of total variance decreases.

The first principal component F1, which accounted for 17.5% of the total variance, was positively correlated with eucalyptol (0.85), the major compound found in EOs of eucalyptus and Chinese yin yang, and with d-limonene (0.70), the main compound found in EOs of bitter orange and pepper. The F2, explaining 16.1% of the total variance, was positively correlated with carvacrol (0.9), the most abundant compound found in oregano EOs.

Based on the PCA analysis, the 14 EOs studied were grouped into six different clusters, which allowed to distinguish six chemotypes as shown in Figure 1. The first group (I) includes eucalyptus and Chinese yin yang, both characterized by a high content of eucalyptol, whereas the second one (II) was made up of bitter orange and pepper, which contains high percentage of D-limonene. Cluster III groups together star anise and fennel, based on the high quantity of anethole. Group IV was clustered by the presence of sesquiterpenes and was composed by cloves, palmarosa, and ylang ylang. Furthermore, it was observed that the EOs of turmeric, lavender, and oregano (Group V) were clustered because of the presence of eucalyptol and o-cymene. In the same way, the similarity between tea tree and Japanese yin yang can be observed (Group VI) due to the high relative percentage of the components eucalyptol and d-limonene.

### 2.2. Antiyeast Activity

Problems with chemical preservatives and the growing demand of consumers for natural food additives have turned the attention to plant-derived natural antimicrobials such as EOs. In fact, EOs could represent an alternative to synthetic preservatives against spoilage due to yeasts and molds. The antiyeast effect of the 14 EOs was tested by the solid medium diffusion method. Table 2 shows the mean diameters of inhibition halos of each EO obtained on the food-spoiling yeast S. cerevisiae. From the results obtained, yeast was resistant to star anise, turmeric, and ylang ylang, as the inhibition halo was completely absent. On the other hand, tea tree, fennel, lavender, pepper and Chinese yin yang showed inhibition halos ranging from 4 to 9 mm. Oregano was the best performing inhibitor of *S. cerevisiae* growth among all the EOs analyzed by displaying a 35-mm inhibition halo (Table 2).

The solid medium diffusion technique was proven to be a useful screening method in order to obtain preliminary data about the antiyeast effect of the EOs. The bitter orange, clove, eucalyptus, oregano, palmarosa, and Japanese yin yang EOs showed growth inhibition halos with diameters equal to or greater than 10 mm. Their minimum inhibitory concentration (MIC) values (Table 3) were showed a more than 90% reduction in the measured absorbance [44].

Amongst the 14 EOs tested, oregano showed the highest antiyeast activity, followed by clove, palmarosa, bitter orange, eucalyptus and Japanese yin yang. Thus, the oregano and clove EOs could be considered as potential antimicrobial agents to be used in the food bioconservation industry.

Oregano was the most active EO against the growth of S. cerevisiae, displaying an MIC of 10 µL/mL (Table 3); this result was in accordance with previous data [6,44]. Antiyeast activity of oregano EO could be associated with the high relative amount of carvacrol, as evidenced by its terpene composition (Table 1). In fact, carvacrol could be absorbed by the double phospholipidic layer of yeasts and could increase the fluidity and permeability of the membrane. Yeast cells, in the presence of carvacrol, have been shown to change the composition of their membrane’s fatty acids as an adaptation mechanism to maintain the correct structure and function of the membrane [6,45].

The antiyeast activity of clove and palmarosa EOs (MIC = 40 µL/mL) was owed to their most abundant components, eugenol and geraniol, respectively [6,45,46,47,48,49].

It is worth noting that the monoterpenes d-limonene, eucalyptol and menthol might be responsible for the antiyeast activity in bitter orange (MIC = 60 µL/mL), eucalyptus (MIC = 60 µL/mL) and Japanese yin yang (MIC = 80 µL/mL) EOs, respectively, as reported by Di Pasqua et al. [45] and Bassolé et al. [50].

### 2.3. Antioxidant Activity

Oxidation is one of the major causes of food degradation, which can occur along the entire food chain. In particular, oxidation is a process that causes unwanted quality changes, organoleptic variation, as well as affect the safety and nutritional value of foodstuffs. It occurs mainly through discoloration, odor generation and off-flavor, or through the formation of potentially toxic substances [2,12]. For this reason, the protection of food from oxidative deterioration is an important goal in food technology. EOs are regarded as GRAS thank to their chemical composition, biological effects and toxicity and are extensively exploited as natural antioxidants to be used in the food sector in contrast to chemical preservatives with known negative effects on human health [2,12]. 

DPPH is a stable free radical widely used to test the free-radical scavenging ability of various EOs. Clove, fennel, lavender, oregano, palmarosa, pepper, star anise, tea tree, turmeric, Chinese yin yang, and ylang ylang EOs were able to inhibit 50% of the radical scavenging activity of DPPH, as can be seen in Table 4. On the contrary, bitter orange, eucalyptus, and Japanese yin yang revealed no antioxidant activity.

The lowest EC_50_ values were found in clove (0.36 ± 0.02 µL/mL) and Chinese yin yang (5.35 ± 0.13 µL/mL), thus they are classified as very strong antioxidants, accordingly to Scherer and Godoy [51] and Cautela et al. [52].

Clove EO was renowned as one of the strongest antioxidants as previously reported by Teixera et al. [22], Misharina and Samusenko [24], Jirovetz et al. [34], and Wei and Shibamoto [53]. The high antioxidant activity of clove could be linked to the presence of eugenol as the EO’s main constituent, revealed by the GC–MS analysis reported in Table 1. This compound is a phenylpropanoid derived from guaiacol and is known to possess antioxidant activity [34,53,54]. Clove EO, due to its high antioxidant activity, could be used as an antioxidant agent in order to prevent natural oxidation and deterioration of food and thus for increasing the shelf life.

Chinese yin yang was a mix of different EOs (*Eucalyptus globulus*, *Cymbopogon citratus*, *Caryophylli aetheroleum*, *Mentha piperita*, *Pinus sylvestris*, *Salvia rosmarinus*, *Lavandula officinalis*, *Foeniculum vulgare*, *Salvia officinalis*, *Illicium verum*, *Mentha arvensis*, and *Abies siberica*) and the very strong activity could be due to the synergistic and additive effects due to the combination of different EOs [15,55,56].

Additionally, oregano and ylang ylang exhibited a strong antioxidant activity and their EC_50_ values were 11.58 ± 0.22 and 12.71 ± 0.17 µL/mL, respectively. Oregano EO was known to possess antioxidant activity due to the presence of carvacrol, as reported in the literature [54], and our study was in accordance with this as this monoterpene phenol resulted to be very abundant (about 70%), as depicted in Table 1. Ylang ylang EO was characterized as having caryophyllene as its main component and so the strong antioxidant activity of EO could be related to presence of this sesquiterpenes, as previously described [57].

Only turmeric displayed a moderate antioxidant activity with 24.99 ± 0.44 µL/mL, while the remaining EOs (fennel, lavender, palmarosa, pepper, star anise, and tea tree) revealed poor antioxidant activity, as reported in Table 4. Overall, clove exhibited the highest antioxidant activity amongst the EOs studied, followed by Chinese yin yang, oregano, and ylang ylang.

## 3. Materials and Methods

### 3.1. Chemicals

2,2-Diphenyl-1-picrylhydrazyl (DPPH) and the MS-grade solvents were purchased from Sigma Chemical Co. (St. Louis, MO, USA). Malt extract, peptone, agar, DMSO and methanol were obtained from Carlo Erba Reagents (Milan, Italy).

### 3.2. Essential Oils

EOs of 13 plants were purchased from different companies, as reported in Table 5.

EOs (clove, eucalyptus, fennel, lavender, oregano, palmarosa, star anise, tea tree, turmeric, Chinese yin yang, Japanese yin yang, and ylang ylang) were mostly obtained by steam distillation. Instead, bitter orange was extracted by means of cold pressing.

Moreover, pepper EO from *Piper niger* was obtained by hydrodistillation in Clevenger-type apparatus according to the European Pharmacopeia method 2005.2812 58 [58]. Briefly, 0.25 kg of pepper leaves were placed in a spherical bottom flask with a volume of 1 L. For optimization of the process, the volume of EO recovered by the Clevenger system was monitored at regulated intervals until the maximum yield was obtained.

### 3.3. GC–MS Analysis

Diluted EO samples (1:100 *v*/*v* in heptane) were analyzed by gas-chromatography–mass spectrometry (GC–MS) using the Trace 1300 GC coupled to the TSQ DUO triple quadrupole mass spectrometer (Thermo Scientific, Walthan, MA, USA) equipped with an electron impact ion source. Samples were injected without derivatization into the DB-5 column, 30-m length, 0.25-mm internal diameter, 0.25-µm film (Thermo Scientific, Walthan, MA, USA) using the 1:10 split mode. The following parameters were used: ionization energy of 70 eV, mass range between 50 and 550 *m/z* and interface temperature of 250 °C. The GC oven temperature was as follows: initial oven temperature of 70 °C and an isotherm for 1 min; subsequently, at 24 °C/min to 180 °C and an isotherm for 2 min, and then reached 280 °C at 50 °C/min where the isotherm was kept for a further 2 min. The carrier gas was helium (He, purity 99.999%) with a constant flow of 1.2 mL/min. 

The acquisition data and the control of the instrument were performed through the Chromeleon Chromatography Data System software, CDS (Thermo Scientific, Walthan, MA, USA). The identification of the GC peaks corresponding to the components of the EOs was based on a direct comparison of the retention times and mass spectral data with those of standard compounds, computer matching with the National Institute of Standards and Technology (NIST) library.

Results were presented as a relative percentage of normalized peak area abundances, without the use of correction factors. The percentage data shown were mean values of two injections.

### 3.4. Antiyeast Activity on Saccharomyces cerevisiae

*Saccharomyces cerevisiae* obtained from the strain collection of the Institute of Research on Terrestrial Ecosystems (IRET) of the National Research Council (CNR) was used to evaluate the antiyeast activity of EOs. Stock culture was maintained at 4 °C on Malt Extract Agar (malt extract 30 g/L; peptone 5 g/L; agar 15 g/L). Inocula were obtained from overnight cultures on MEA plates at 28 °C. *S. cerevisiae* was grown in malt extract (ME 30 g/L; peptone 5 g/L) for 24 h in an orbital shaking incubator at 120 rpm at 28 °C [59]. The growth was monitored both by measuring the absorbance at 600 nm and by counting on plates (CFU/mL).

#### 3.4.1. Solid Medium Diffusion Method

A solid medium diffusion procedure using wells in dishes was used to determine the antiyeast activity of all EOs [22,44]. For this, 1 mL of *S. cerevisiae* suspension with a concentration of 10^6^ CFU/mL was uniformly spread on a sterile MEA petri dish (diameter 9 cm). After inoculum absorption by agar, wells were made using sterile glass tubes (diameter 6 mm) which were filled with 10 µL of each EO. The disc radius was not included. Negative controls were prepared with only 10 µL of DMSO. Petri dishes were incubated at 28 °C for 48 h; antiyeast activity was evaluated by measuring the diameter of the growth inhibition halos and was expressed in millimeters. All determinations were carried out in triplicate.

#### 3.4.2. Minimum Inhibitory Concentration

EOs showing growth inhibition, as clear white zone with diameters equal to or greater than 10 mm with the solid medium diffusion method, were considered to determine the minimum inhibitory concentration (MIC) using a liquid medium. The MIC was defined as the lowest concentration of an EO that resulted in a reduction of >90% in the measured absorbance [15,22,44,60].

The microplate bioassay was used to study the antiyeast activity of EOs. The 24-well plates were prepared by dispensing into each well 1.8 mL of Malt Extract broth and 0.2 mL of yeast inoculum with a final concentration of 10^6^ CFU/mL. An aliquot (20 µL) of each EO (with concentrations ranging from 0.1 to 1000 µL/mL) was transferred into a well. Negative controls were prepared adding only 20 µL of DMSO to ME medium. The microplates were sealed and incubated on a plate shaker (100 rpm) at 28 °C for 48 h. The yeast growth was evaluated by measuring the absorbance at 600 nm.

### 3.5. Antioxidant Activity

The radical scavenging activity (RSA) of EOs was evaluated by 2,2′-diphenyl-1-picrylhydrazyl (DPPH) assay according to the procedure of Blois [61]. Briefly, 150 μL of each EOs (with a concentration ranging from 0.1 to 1000 µL/mL) were mixed with 1.35 mL of 60-μM DPPH methanolic solution. The absorbance reduction at 517 nm of the DPPH was determined continuously for 60 min. The RSA was calculated as a percentage of DPPH discoloration, using the following equation:

(1) $$\%\mathrm{RSA}=\;\frac{\left(A_{\mathrm{DPPH}}\;-\;A_{s}\right)}{A_{\mathrm{DPPH}}}\;\times 100$$

where A_S_ is the absorbance of the solution when the EO was added and A_DPPH_ is the absorbance of the DPPH solution, as reported [62]. The extract concentration (EC) necessary to achieve 50% radical DPPH inhibition (EC_50_) was obtained by plotting the RSA percentage as a function of EOs’ concentrations and was expressed as microliters per milliliter (µL/mL).

Moreover, in order to classify the EOs, the antioxidant activity index (AAI) was determined as follows:

(2) $$\mathrm{AAI}=\;\mathrm{DPPH}\;\mathrm{concentration}\;\mathrm{in}\;\mathrm{reaction}\;\mathrm{mixture}/{\mathrm{EC}}_{50}$$

EOs were categorized as showing poor antioxidant activity (AAI < 0.5), moderate (0.5 < AAI < 1.0), strong (1.0 < AAI < 2.0) and very strong (AAI > 2.0), as reported by Scherer and Godoy [51] and Cautela et al. [52].

### 3.6. Statistical Analysis

Samples were analyzed in triplicate and all results were expressed as mean ± standard deviation (SD). Means, SD, calibration curves and linear regression analyses (R^2^) were determined using Microsoft Excel 2013 (Microsoft Corporation, Redmond, WA, USA). The multivariate analysis was performed applying Principal Component Analysis (PCA) through XLSTAT Statistical Software using Microsoft Excel 2013. 

## 4. Conclusions

From analyzing the chemical composition of the 14 EOs, bitter orange was found to consist mainly of monoterpenes while tea tree, eucalyptus, lavender, palmarosa, Chinese yin yang, and Japanese yin yang were characterized by high amounts of monoterpenoids and oxygenated monoterpenes. Sesquiterpenes, on the other hand, were abundant in ylang ylang, while star anise, fennel, clove, and oregano were rich in phenylpropanoids. Turmeric and pepper EOs exhibited a more heterogeneous chemoprofile and could not be associated with a prevalent terpenic class.

Overall, the 14 EOs studied were classified into six different chemotypes according to their chemical compositions and their relative abundances. The exploratory PCA technique allowed us to visualize, by reducing the dimension of the original data, and provide phytochemical relationships among all the EOs studied. Summarizing our results, amongst the 14 EOs studied, clove showed the highest antioxidant activity with an EC_50_ of 0.36 µL/mL, followed by Chinese yin yang, oregano and ylang ylang. Moreover, oregano had the greatest antiyeast properties, inhibiting the growth of *S. cerevisiae* with an MIC of 10 µL/L, followed by clove, palmarosa, bitter orange, eucalyptus and Japanese yin yang EOs. Therefore, considering the EOs studied here, clove for its high antioxidant activity and oregano for its great antiyeast activity may have potential uses in the food and beverage sectors to increase shelf life and avoid deterioration.

## Figures and Tables

**Table 1:** Chemical composition of 14 essential oils (Eos) expressed as relative concentrations (% = peak area percent; RT = Retention Time).

RT (min)	Compound	Bitter Orange (%)	Clove (%)	Eucalyptus (%)	Fennel (%)	Lavender (%)	Oregano (%)	Palmarosa (%)	Pepper (%)	Star Anise (%)	Tea Tree (%)	Turmeric (%)	Chinese Yin Yang (%)	Japanese Yin Yang (%)	Ylang Ylang (%)
5.37	β-Phellandrene	0.206			0.212				0.937		0.079			0.323	
5.53	β-Pinene	0.602		0.183	0.825				0.784		0.141	0.159	2.929	11.953	0.074
5.54	Amyl vinyl carbinol					0.270	0.399								
5.75	Ethyl amyl ketone					0.975	0.102								
5.86	β-Myrcene	0.971		0.111	0.509		1.567	0.383	2.904		0.105		0.857		0.092
6.03	Amyl ethyl carbinol					0.306								0.127	
6.30	(−)-α-thujene				2.462		0.200		3.072		0.327	10.683	0.534		
6.51	3-Carene					0.267				0.210		0.222	5.412		
6.77	α-Terpinolene										7.097				
6.86	Menthene										0.055				4.464
6.91	(+)-4-Carene						1.396								
7.02	o-Cymene			7.865	1.615	0.443	14.002		23.928	0.340	11.591	2.744	2.806	0.084	
7.17	d-Limonene	93.947		0.904	9.004	0.228	0.358	0.159	21.281	1.152	1.992	0.738	8.937	3.127	
7.28	Eucalyptol			89.338		3.578	0.181		0.157	0.302	2.201	2.609	34.462	0.099	0.301
7.49	2-Norpinene							0.169							
7.68	Lavender lactone					0.400									
7.87	β-o-Cymene							0.380							
8.25	γ-Terpinene				0.326		7.504				17.225		1.264		
8.29	α-Pinene				0.225							0.331			
8.81	*cis*-Linaloloxide					5.848								0.058	
9.30	Fenchone				18.779										
9.32	Isoterpinolene											0.407	1.294		
9.35	Linalool oxide					5.203									1.695
9.68	Linalool	0.243		0.079		23.294	1.351	2.548		0.300			0.511	0.218	9.471
9.75	Perillen								0.072						
9.89	δ-Thujone												0.262		
10.19	α-Fenchol					0.898					0.066				
10.4	trans-*p*-Mentha-2,8-dienol	0.243													
10.52	3-Octanol-acetate					0.138									
10.8	Limonene oxide, *cis*-	0.559									0.207				
10.87	*p*-Mentha-2,8-dien-1-ol	0.370													
10.94	Limonene oxide, *trans*-	0.517													
11.00	Isopinocarveol			0.180							0.642				
11.14	Camphor				0.364	4.031					0.057		1.049		
11.18	Thujol					0.101					0.323			1.217	
11.39	Citronellal												7.528		
11.43	l-Menthone												2.357	33.836	
11.73	*p*-Menthan-3-one												1.275		
11.77	Menthol													35.623	
11.86	endo-Borneol					2.377	0.183								1.755
11.92	Carvenone					0.475			0.123						
12.10	*p*-Menthan-1-ol												4.582		
12.13	Neoisoisopulegol													1.439	
12.16	Terpinen-4-ol			0.279		1.190	0.446		0.087	0.138	47.525		0.521		
12.34	*trans*-3-Caren-2-ol					0.079			0.054						
12.42	Cryptone					0.199			0.418						
12.57	α-Terpineol						0.071			0.178					0.120
12.58	Terpineol	0.153		0.868		3.259					8.537		1.745	7.064	
12.8	Estragole				14.115					0.256					0.147
12.82	Teresantalol	0.125													
12.85	Verimol H										0.081		0.079		
12.88	Sabinol	0.157							0.588						
13.13	d-Verbenone			0.060		0.088									
13.39	*trans*-Carveol	0.393													
13.48	Geranyl vinyl ether					0.203									
13.56	Isoascaridol										0.055				
13.62	Isobornyl formate					0.544									
13.63	Nerol	0.267						0.362					1.627		
13.75	Fenchyl acetate				0.114										
13.94	Pulegone					0.098			0.120				0.097		
14.07	(−)-Carvone	0.320		0.132			0.133		0.075						
14.14	Carvotanacetone								0.076						
14.29	*cis*-Anethole				0.097					0.163					
14.33	Linalyl acetate	0.484													
14.36	Piperitone													0.208	
14.37	*p*-Anisaldehyde				0.466	30.273				8.881					
14.4	Geraniol							82.587					2.416	0.332	0.802
14.48	Benzaldehyde									0.338					
14.75	α-Citral							0.825							
14.77	trans-Ascaridol glycol					0.175					0.601				
14.88	Phellandral					0.470			0.179						
15.16	Bornyl acetate					0.222			0.142				9.963		
15.25	Anethole	0.143			50.826					74.997	0.020				
15.26	Lavandulyl acetate					2.274									
15.33	Menthol acetate												0.836	3.503	
15.57	Linalyl formate							0.145							
15.76	Carvacrol						69.641		0.264						
15.95	Carvomenthol								0.951						
16.25	Hexyl tiglate					0.207									
16.43	Elemene														0.090
16.48	Hotrienol					2.476									
16.54	Limonene-diol	0.163													
16.71	α-Copaene								0.127						
16.72	α-Cubebene														0.119
17.03	Eugenol		82.723										2.538		0.348
17.05	Neryl acetate					0.262		0.088							
17.25	Ylangene														0.460
17.31	trans-Ascaridol glycol					0.451									
17.37	Copaene		0.268						0.306	6.792			0.144		1.488
17.49	Geranyl acetate					0.514		10.122					0.159		8.276
17.56	(−)-β-Bourbonene					0.244								0.092	
17.57	*p*-Acetonylanisole				0.061					5.613					
17.69	*cis*-Copaene														0.199
17.73	β-Elemene								0.647				0.508		0.501
17.74	Farnesol acetate							0.058							
17.82	β-Copaene														0.060
17.84	Ascaridole epoxide					0.340									
18.07	*threo*-Anethole glycol									0.056					
18.13	Isoledene								1.206						
18.20	Limonene oxide, *cis*-					0.643									
18.25	trans-α-Bergamotene									0.055					
18.37	α-Santalene					0.444									
18.40	Caryophyllene		5.114				2.103	1.789	2.823	0.060	0.723	0.370	1.482	0.294	15.541
18.45	α-Bulnesene														0.084
18.55	8-Hydroxy-carvotanacetone					0.084									
18.70	*cis*-α-Bergamotene									0.168					
18.83	γ-Patchoulene												0.090		
18.94	(±)-Thujpsadiene											0.447			
19.15	Humulene		0.355					0.141	1.012				0.214		4.728
19.29	Alloaromadendrene								0.523		0.090				
19.6	Muurolene								0.616						1.882
19.71	*cis*-β-Copaene					0.201			0.643				0.320		19.461
19.72	α-Curcumene											2.745			
19.73	Mullilam diol					0.164									
19.99	δ-Curcumene											2.281			
20.00	7-epi-Sesquithujene											0.218			
20.02	Germacrene D								0.822						0.915
20.05	(+)-Ledene										0.080				
20.07	γ-Elemene														0.622
20.11	α-Muurolene								1.575				0.121		1.184
20.21	α-Farnesene								0.407						8.064
20.27	β-Bisabolene					0.259	0.118					0.596			
20.41	γ-Muurolene					0.378			1.628						
20.42	Isogermacrene D														1.057
20.43	Cubenol					0.513							0.120		
20.45	10-epi-Cubebol								0.271						
20.52	Teresantalol					0.201									4.016
20.59	δ-Amorphene								3.855				0.593		
20.60	Sesquisabinene											2.170			
20.65	Epizonarene														0.110
20.66	Eugenol acetate		10.281												
21.01	α-Calacorene								0.098						
21.13	Elemyl acetate								2.673				0.366		
21.54	Palustrol								0.513						
21.67	trans-pyran Linalool oxide					0.868									
21.69	Germacrenol								1.300						
21.74	(−)-Spathulenol								1.066						
21.88	Caryophyllene oxide		1.197			3.148	0.224	0.244	1.195			0.372			0.193
21.90	(−)-Globulol										0.126				
22.01	(±)-Dihydro-*ar*-turmerone											0.444			
22.03	Epiglobulol								0.909		0.053				
22.04	Epi-Cadinol														0.422
22.25	Ledol								0.588						
22.32	*cis*-Nuciferol											0.708			
22.40	Humulene epoxide		0.061			0.272			0.473			0.159			
22.57	Junenol														0.300
22.59	α-Corocalene								0.060						
22.72	10-epi-Cubenol														0.254
22.79	10-epi-γ-Eudesmol								1.065						
22.91	(*Z*)-γ-Atlantone								0.696			0.601			
22.97	τ-Muurolol								1.243						0.414
22.98	τ-Cadinol					0.063									0.994
23.06	Cubebene								0.349						
23.17	7-epi-β-Eudesmol								2.211						
23.22	α-Cadinol								2.944						0.080
23.28	(*Z*)-α-Atlantone								0.109			0.200			
23.38	α-Turmerone											28.801			
23.46	*ar*-Tumerone											29.341			
23.57	Isoaromadendrene epoxide					0.218									
23.95	6-epi-Shyobunol								3.507						
24.07	Curlone											12.402			
24.39	*trans*-Farnesol														1.953
24.77	Oplopanone								0.330						
25.23	Ascabiol														0.492
25.32	*trans*-*Z*-α-Bisabolene epoxide					0.064			1.034						
25.42	*cis*-*Z*-α-Bisabolene epoxide					0.077									
25.44	Cubenol								1.126						
25.47	(*E*)-Atlantone											0.252			
25.50	(−)-Spathulenol								0.171						
26.11	Isoaromadendrene epoxide								3.259						
26.28	(+)-Spathulenol								0.191						6.694
27.12	α-Isonootkatol								0.062						
27.24	Platambin								0.088						
27.56	Ylangenol								0.064						

**Table 2:** Results of antiyeast activity of 14 essential oils using the solid medium diffusion assay.

Essential Oil	Mean Diameter (mm)
Bitter orange	15
Clove	17
Eucalyptus	15
Fennel	5
Lavender	7
Oregano	35
Palmarosa	15
Pepper	4
Star anise	N.D.
Tea tree	8
Turmeric	N.D.
Chinese yin yang	9
Japanese yin yang	13
Ylang ylang	N.D.

N.D. = inhibition zone diameter not detected.

**Table 3:** Minimum inhibitory concentration values (in μL/mL) of bitter orange, clove, eucalyptus, oregano, palmarosa and Japanese yin yang EOs.

Essential Oil	MIC (µL/mL)
Bitter orange	60
Clove	40
Eucalyptus	60
Oregano	10
Palmarosa	40
Japanese yin yang	80

**Table 4:** Antioxidant activity of EOs measured by 2,2-diphenyl-1-picrylhydrazyl (DPPH) method and expressed as EC_50_.

Essential Oil	EC_50_ (µL/mL)	AAI
Bitter orange	N.D.	N.D.
Clove	0.36 ± 0.02	59.17
Eucalyptus	N.D.	N.D.
Fennel	90.86 ± 0.14	0.23
Lavender	665.54 ± 0.50	0.03
Oregano	11.58 ± 0.22	1.84
Palmarosa	950.52 ± 0.71	0.02
Pepper	62.10 ± 0.23	0.34
Star anise	500.57 ± 0.33	0.04
Tea tree	54.81 ± 0.24	0.39
Turmeric	24.99 ± 0.44	0.85
Chinese yin yang	5.35 ± 0.13	3.98
Japanese yin yang	N.D.	N.D.
Ylang ylang	12.71 ± 0.17	1.68

AAI = antioxidant activity index; N.D. = antioxidant activity not detected. Values presented as mean ± standard deviation.

**Table 5:** Botanical, geographical, and commercial sources of EOs.

Essential Oil	Species	Part of Plant	Country	Company
Bitter orange	*Citrus aurantium*	Peels	Ivory Coast	Essenthya
Clove	*Eugenia caryophyllata*	Buds	Sri Lanka	Primavera
Eucalyptus	*Eucalyptus globulus*	Leaves and twigs	Hungary	Phoenix Pharma
Fennel	*Foeniculum vulgare*	Seeds	Italy	Primavera
Lavender	*Lavandula officinalis*	Flowers	Bulgaria	Primavera
Oregano	*Origanum vulgare*	Flowering plants	Spain	Primavera
Palmarosa	*Cymbopogon martini*	Flowering plants	India	Essenthya
Star anise	*Illicium verum*	Fruits and seeds	Vietnam	Primavera
Tea tree	*Melaleuca alternifolia*	Leaves and twigs	Australia	Naturando
Turmeric	*Curcuma longa*	Rhizomes	Madagascar	Essenthya
Chinese yin yang	*Mix (Eucalyptus globulus, Cymbopogon citratus, Caryophylli aetheroleum, Mentha piperita, Pinus sylvestris, Salvia rosmarinus, Lavandula officinalis, Foeniculum vulgare, Salvia officinalis, Illicium verum, Mentha arvensis, Abies siberica)*	–	Austria	Best of Nature
Japanese yin yang	*Mentha arvensis*	Whole plants	Austria	Best of Nature
Ylang ylang	*Cananga odorata*	Flowers	Madagascar	Essenthya
